# Covid-19, primary care, and community surveillance in vulnerable areas: lessons learned from the results of the TQT-Covid-19 Study

**DOI:** 10.11606/s1518-8787.2026060S1ed

**Published:** 2026-05-01

**Authors:** Alexandre Dias Porto Chiavegatto, Bruno Pereira Nunes

**Affiliations:** I Universidade de São Paulo. Faculdade de Saúde Pública. Departamento de Epidemiologia. São Paulo, SP, Brasil; II University of Illinois Urbana-Champaign. Department of Health and Kinesiology. Urbana, IL, United States

The TQT-Covid-19 supplement, published in this issue of the Revista de Saúde Pública, stems from a fundamental question for contemporary public health. It begins by comparing two approaches to responding to a pandemic. On the one hand, you have a population-based approach, with coordination of care through primary health care (PHC) in vulnerable areas, large-scale testing, and active surveillance in partnership with the community. On the other hand, there is a predominantly clinical strategy, centered on the hospital network.

The studies gathered here, based on the results of the TQT-Covid-19 Study in Salvador (BA) and Rio de Janeiro (RJ), offered one of the most comprehensive panels ever produced in the country on how highly vulnerable populations, PHC services, and health care workers responded to the Covid-19 pandemic and how the results can guide preparation for future health emergencies.

Some results of the study deserve highlighting. Regarding individual protective behaviors, the article on the adoption of preventive behaviors against Covid-19 by users of health units shows high (although not universal) prevalences of respiratory etiquette and hand hygiene (ranging between 79.1% and 84.9%), in addition to greater adherence to social distancing by older people who were not in paid employment and who received booster doses of Covid-19 and influenza vaccines. The results point to the role of immunization strategies, health communication, and sociodemographic characteristics in maintaining protective behaviors among populations in situations of socioeconomic vulnerability^1^.

In health surveillance, the study analyzing the strategies used to organize the demand for testing showed relevant results based on indicators of testing, quarantine, contact tracing, and telemonitoring^2^. In 12 months, more than 12,000 people were reached and almost 12,000 tests were performed. Significant positivity rates were observed, higher in Salvador, and complete vaccination coverage above 90%, while operational limitations were identified in routinely incorporating telemonitoring and contact tracing, which are critical components for mitigating new epidemic waves in contexts of high transmission. It is worth recalling that Brazil has done little to promote case surveillance as a national policy. Even in this unfavorable context, the results indicate the benefits of active surveillance coordinated by PHC for epidemic control.

No consistent analysis of the pandemic response in PHC is complete without considering the effects on health care workers, who sustain the system on a daily basis. The qualitative article on the mental health of health care professionals in Manguinhos^3^ powerfully describes how the constant fear of death, work overload, lack of adequate personal protective equipment, feelings of powerlessness in decision-making, and the absence of structured mental health support led to situations of intense suffering.

Among community health workers, the study also highlighted the perception of exclusion in relation to other categories of health professionals, which makes these workers simultaneously central to the territorial strategy, yet invisible in institutional protection. The same study argues for the need to incorporate, from the beginning of a health crisis, actions for psychological protection, clear rules for the division of tasks, and effective access to mental health care for all professional categories.

Alongside these results, the supplement also brings together new evidence on the acceptability of Covid-19 self-testing among highly vulnerable users, the ability to comply with home isolation in households with high population density, health care professionals’ perceptions of testing in primary care units, the impact of the pandemic on the precariousness of work in PHC, and the factors associated with the use of unproven medications for the prevention of Covid-19. These findings, among others, are highly relevant to the literature, health system management, and the adoption of effective health policies for periods with and without pandemics, but especially for preparing for and responding to future epidemics. Brazil, unfortunately, was not a good example of a national response to the Covid-19 pandemic, but the strategies presented in this supplement show that managers, health care workers, and researchers were able to innovate, through public health approaches, even in an unfavorable context, to improve care and guarantee the right to health for all during a pandemic.

Taken together, the articles in this supplement demonstrate that discussing pandemic preparedness without considering PHC in vulnerable areas is an ineffective decision with high human cost. It is in the connection between living conditions, the organization of health work, testing and surveillance strategies, and the protection of the mental health of health care workers that the capacity of a system to respond to the next health emergency is defined. We hope that this supplement will be widely disseminated not only as a record of the Brazilian response to Covid-19 in vulnerable contexts, but also as a reference for designing policies for community surveillance, reorganizing PHC, and protecting the health care workforce in a reality of possible recurring health emergencies.
