# Supplementary material for: Covid-19, primary care, and community surveillance in vulnerable areas: lessons learned from the results of the TQT-Covid-19 Study
Source: Rev Saude Publica. 2026 May 1;60(Suppl 1):e1s. doi: 10.11606/s1518-8787.2026060S1ed (PMC13143265; doi:10.11606/s1518-8787.2026060S1ed)
Supplement: Translation

# Translation: Covid-19, atenção primária e vigilância comunitária em territórios vulnerabilizados: lições aprendidas com os resultados do Estudo TQT-Covid-19

**Authors:** Alexandre Dias Porto Chiavegatto, Bruno Pereira Nunes

**Affiliations:** I Universidade de São Paulo. Faculdade de Saúde Pública. Departamento de Epidemiologia. São Paulo, SP, Brasil; II University of Illinois Urbana-Champaign. Department of Health and Kinesiology. Urbana, IL, United States of America

O suplemento TQT-Covid-19, publicado nesta edição da Revista de Saúde Pública, nasce de uma pergunta fundamental para a saúde pública contemporânea. Ele parte da comparação de dois caminhos de resposta a uma pandemia. De um lado, uma abordagem populacional, com coordenação do cuidado pela atenção primária à saúde (APS) em territórios vulnerabilizados, testagem em larga escala e vigilância ativa em parceria com a comunidade. Do outro, uma estratégia predominantemente clínica, centrada na rede hospitalar.

Os estudos aqui reunidos, baseados nos resultados do Estudo TQT-Covid-19 em Salvador (BA) e no Rio de Janeiro (RJ), ofereceram um dos painéis mais abrangentes já produzidos no país sobre como populações em alta vulnerabilidade, serviços de APS e trabalhadores da saúde responderam à pandemia de Covid-19 e como os resultados podem orientar a preparação para futuras emergências sanitárias.

Alguns resultados do estudo merecem destaque. Em relação aos comportamentos individuais de proteção, o artigo sobre a adoção de comportamentos preventivos de Covid-19 por usuários de unidades de saúde mostra prevalências elevadas (embora não universais) de etiqueta respiratória e higienização das mãos (variando entre 79,1 % e 84,9%), além de maior adesão ao distanciamento social por pessoas mais velhas, que não estavam em atividade remunerada e que receberam doses de reforço das vacinas para Covid-19 e *influenza*. Os resultados apontam para o papel das estratégias de imunização, da comunicação em saúde e das características sociodemográficas na manutenção de comportamentos protetores das populações em situação de vulnerabilidade socioeconômica^1^.

Na vigilância em saúde, o estudo que analisa as estratégias utilizadas para organizar a demanda para testagem evidenciou resultados relevantes com base nos indicadores de testagem, quarentena, rastreamento de contatos e telemonitoramento^2^. Em 12 meses, mais de 12 mil pessoas foram alcançadas e quase 12 mil testes realizados. Foram observadas taxas de positividade expressivas, maiores em Salvador, e cobertura vacinal completa acima de 90%, ao mesmo tempo que foram identificados limites operacionais para incorporar de forma rotineira o telemonitoramento e o rastreamento de contatos, que são componentes críticos para mitigar novas ondas epidêmicas em contextos de alta transmissão. Vale lembrar que o Brasil pouco fomentou a vigilância de casos como política nacional. Mesmo nesse contexto desfavorável, os resultados indicam os benefícios da vigilância ativa coordenada pela APS para controle de epidemias.

Nenhuma análise consistente da resposta à pandemia em APS é completa sem considerar os efeitos sobre os trabalhadores em saúde, os quais sustentam o sistema no cotidiano. O artigo qualitativo sobre saúde mental de profissionais de saúde em Manguinhos^3^ descreve de forma contundente como o medo constante da morte, a sobrecarga de trabalho, a falta de equipamentos de proteção individual adequados, a sensação de impotência decisória e a ausência de suporte estruturado em saúde mental levaram a situações de sofrimento intenso.

Entre agentes comunitários de saúde, o estudo destacou ainda a percepção de exclusão em relação à categoria de profissionais de saúde, o que torna esses trabalhadores simultaneamente centrais para a estratégia territorial, porém invisíveis na proteção institucional. O mesmo estudo defende a necessidade de incorporar, desde o início de uma crise sanitária, ações de proteção psíquica, regras claras de divisão de tarefas e acesso efetivo a cuidado em saúde mental para todas as categorias profissionais.

Ao lado desses resultados, o suplemento reúne também novas evidências sobre a aceitabilidade do autoteste para Covid-19 entre usuários em alta vulnerabilidade, a capacidade de cumprir isolamento domiciliar em domicílios com alta densidade de moradores, as percepções de profissionais sobre a testagem em unidades básicas, o impacto da pandemia na precarização do trabalho em APS e os fatores associados ao uso de medicamentos sem comprovação científica para prevenção da Covid-19. Essas descobertas, entre outras, são muito relevantes para a literatura, a gestão do sistema de saúde e a adoção de políticas de saúde efetivas para períodos com e sem pandemias, mas, principalmente, para o preparo e o enfrentamento de futuras epidemias. O Brasil, infelizmente, não foi um bom exemplo de enfrentamento nacional da pandemia de Covid-19, mas as estratégias apresentadas neste suplemento mostram que gestores, trabalhadores do sistema de saúde e pesquisadores foram capazes de inovar, por meio de abordagens de saúde pública, mesmo em um contexto desfavorável, para melhorar o cuidado e garantir o direito à saúde para todos durante uma pandemia.

Em conjunto, os artigos deste suplemento demonstraram que falar em preparação para pandemias sem olhar para a APS em territórios vulnerabilizados é uma decisão inefetiva e com alto custo humano. É na ligação de condições de vida, organização do trabalho em saúde, estratégias de testagem e vigilância e proteção da saúde mental dos trabalhadores que se define a capacidade de um sistema responder à próxima emergência de saúde. Esperamos que este suplemento seja amplamente divulgado não apenas como um registro da resposta brasileira à Covid-19 em contextos de vulnerabilidade, mas também como uma referência para o desenho de políticas de vigilância comunitária, reorganização da APS e proteção da força de trabalho em saúde, numa realidade de possíveis emergências sanitárias recorrentes.
